# Molecular mechanism of berberine in ameliorating leptin resistance and mitochondrial dysfunction through the TRIB1-C/EBPα axis in obesity

**DOI:** 10.1186/s13020-025-01296-7

**Published:** 2026-01-26

**Authors:** Xuelian Zhang, Chenyang Zhang, Xiangrui Meng, Jianyuan Tang

**Affiliations:** 1 TCM Prevention and Treatment of Metabolic and Chronic Diseases Key Laboratory of Sichuan Province, Hospital of Chengdu University of Traditional Chinese Medicine, Chengdu, 611137 China; 2 Clinical School of Medicine, Chengdu University of Traditional Chinese Medicine, Chengdu, 611137 China; 3 Beijing Key Laboratory of Diabetes Research and Care, Department of Endocrinology, Beijing Diabetes Institute, Beijing Tongren Hospital, Capital Medical University, Beijing, 100730 People’s Republic of China

**Keywords:** Berberine, TRIB1, Leptin signaling, Mitochondrial fission/fusion, Energy expenditure, Metabolic remodeling

## Abstract

Hyperleptinemia and mitochondrial dysfunction in obesity form a vicious cycle, underscoring the need for targeted interventions. This study suggests that berberine reduces leptin synthesis and improves leptin resistance by upregulating adipose tissue pseudokinase TRIB1 expression, promoting COP1-mediated C/EBPα ubiquitination and degradation, enhancing STAT3 phosphorylation, and suppressing SOCS3 expression. Meanwhile, TRIB1 appears to mediate the remodeling of mitochondrial dynamics by increasing the expression of fusion proteins MFN1 and L-OPA1, inhibiting the activity of the fission protein DRP1, reversing mitochondrial fragmentation, improving respiratory metabolic capacity, and thereby enhancing brown adipose tissue (BAT) thermogenesis. In TRIB1 knockout mice, the dual effects of berberine—central reduction of high-fat diet intake and peripheral promotion of lipolysis and thermogenesis—were largely abolished. Collectively, these findings support a model in which TRIB1 serves as a critical mediator through which berberine coordinates leptin signaling and mitochondrial function, providing mechanistic insight that may inform future strategies for obesity intervention.

**Graphical Abstract:**

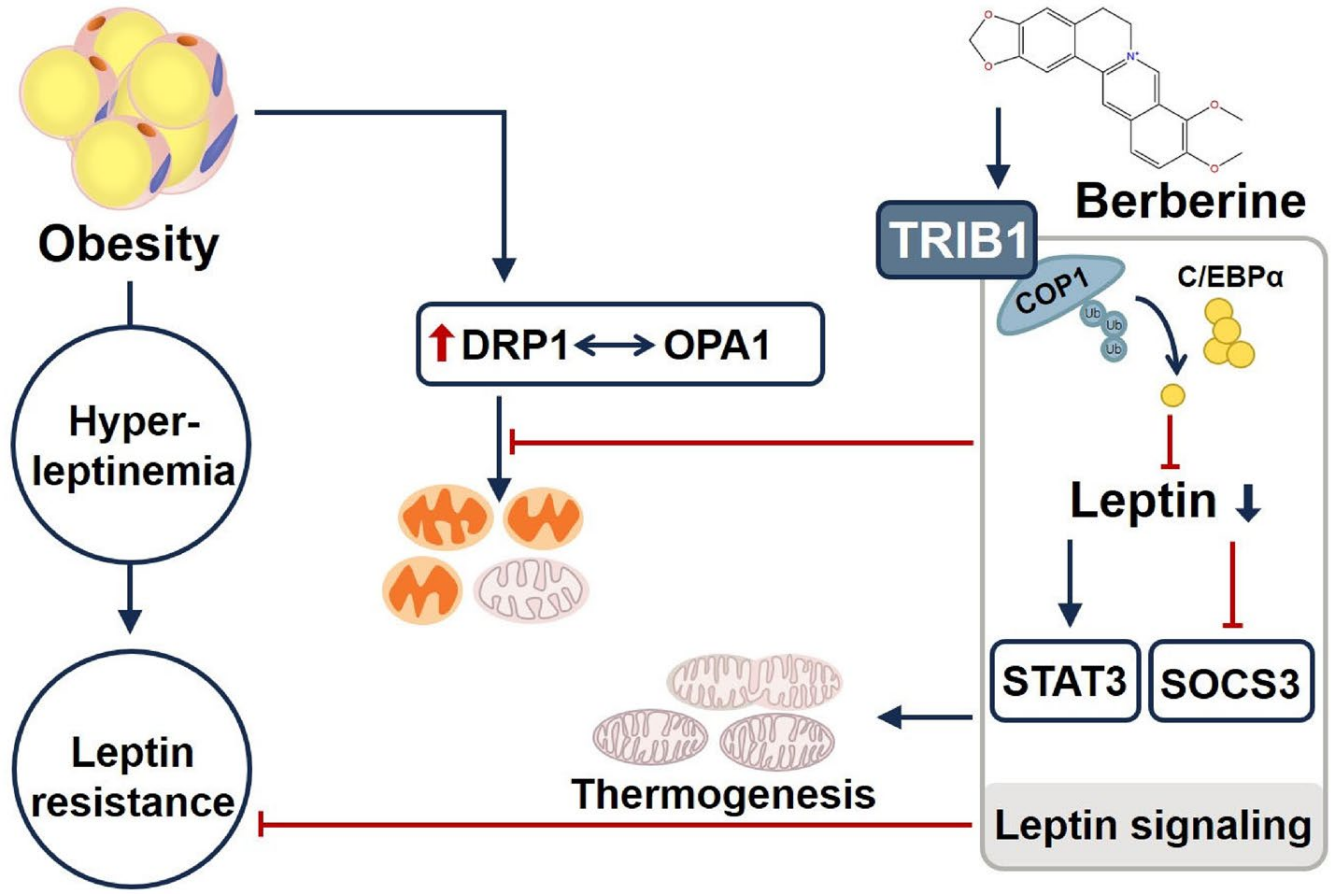

**Supplementary Information:**

The online version contains supplementary material available at 10.1186/s13020-025-01296-7.

## Introduction

The global obesity epidemic drives a cascade of lipid metabolism disorders, including hyperlipidemia, non-alcoholic fatty liver disease (NAFLD), and leptin resistance. These pathologies arise not only from ectopic lipid accumulation but are also closely linked to chronic inflammation and energy imbalance. Emerging evidence has identified the pseudokinase TRIB1 as an important regulator of lipid metabolism. Genome-wide association studies have reported significant associations between the TRIB1 locus and multiple circulating lipid traits [1, 2]. TRIB1 is thought to modulate lipid homeostasis through several mechanisms: acting as a molecular scaffold that recruits the COP1 ubiquitin ligase to promote C/EBPα ubiquitination and degradation [3], thereby limiting de novo hepatic lipogenesis [4]; regulating LDL receptor (LDLR) expression to influence plasma cholesterol clearance [5]; and restoring cholesterol homeostasis under circadian rhythm disruption, as shown by recent findings. In liver-specific *Bmal1* knockout models, TRIB1 overexpression reduces PCSK9 levels, increases LDLR protein, and attenuates hypercholesterolemia [6]. Previous work also suggests that TRIB1 deficiency impairs mitochondrial respiratory chain complexes and suppresses the lipid oxidative capacity of brown adipose tissue (BAT) [7]. Together, these findings support a model in which TRIB1 participates in the coordinated control of systemic lipid metabolism.

Leptin, a core adipokine, maintains energy homeostasis through dual mechanisms: it binds hypothalamic arcuate nucleus leptin receptors, activating the JAK2-STAT3 pathway to promote POMC neuron activity and inhibit appetite-related neuropeptides [8]; and it enhances sympathetic output, stimulating adipose tissue β3-adrenergic receptors (β3-AR), and inducing lipolysis and UCP1-mediated thermogenesis via the cAMP-PKA pathway [9]. In obesity, however, persistent hyperleptinemia induces negative feedback: inflammatory cytokines such as TNF-α and IL-6 elevate SOCS3 expression, limiting STAT3 phosphorylation and nuclear translocation, while PTP1B dephosphorylates JAK2. These processes together contribute to leptin resistance [10]. Notably, leptin resistance and mitochondrial dysfunction form a vicious cycle. Reduced sympathetic tone weakens β3-AR signaling, leading to an imbalance in mitochondrial dynamics regulators: DRP1-mediated fission is enhanced [11], while MFN1/L-OPA1-mediated fusion is impaired, causing mitochondrial fragmentation, respiratory chain damage, reduced oxidative phosphorylation efficiency, and ultimately diminished BAT thermogenic capacity.

Berberine, a protoberberine alkaloid derived from *Coptis chinensis*, has been studied for metabolic benefits that extend beyond classical AMPK activation. Prior studies indicate that berberine can reduce circulating triglycerides by increasing hepatic LDLR expression and suppress lipogenesis through inhibition of SREBP1c [12]. Berberine has also been reported to lower circulating leptin and restore hypothalamic STAT3 phosphorylation in obese models, although the upstream regulators and tissue specificity of these effects remain incompletely defined, and it is unclear whether TRIB1 is required [13]. Given that TRIB1 has been implicated in COP1-dependent C/EBPα ubiquitination and degradation, and in the regulation of mitochondrial fusion proteins such as MFN1 and OPA1, we hypothesized that berberine may act, at least in part, by upregulating TRIB1 in adipose tissue. We further posited that TRIB1 could facilitate C/EBPα ubiquitination, reduce leptin synthesis, and alleviate mitochondrial fragmentation associated with DRP1 activity, thereby improving leptin signaling and mitochondrial function in obesity. To explore this possibility, we used TRIB1 knockout mice and 3T3-L1 adipocytes to examine the TRIB1–C/EBPα–leptin axis and mitochondrial dynamics under berberine treatment.

## Methods

### Animals and treatments

Six-week-old C57BL/6J male mice were purchased from Nanjing Gempharmatech Co. Ltd. (Nanjing, China). CRISPR/Cas9-mediated TRIB1-knockout mice were produced by Cyagen Biosciences Inc. (Suzhou, China), the breeding and genotyping strategies for TRIB1-KO mice were performed as described previously [7]. All animal experiments were performed in accordance with the guidelines of the National Institutes of Health Guide for the Care and Use of Laboratory Animals (NIH Publication No. 85-23, revised 1996) and were approved by the Experimental Laboratory Animal Committee of the Institute of Medicinal Plant Development, Peking Union Medical College. The mice were maintained under standard laboratory conditions (room temperature at 22 °C, humidity of 60% with a 12 h light/dark cycle) and were allowed free access to food and water.

After 1 week of adaptation, a total of 24 mice were randomly allocated to four groups, with six mice in each group (n = 6): (1) Control group, mice were fed control diet; (2) HFD group, mice were fed high fat diet; (3) Orlistat group, HFD plus orlistat 30 mg/kg; (4) Berberine group, HFD plus berberine 100 mg/kg. Based on our preliminary data and previous reports demonstrating efficacy in obese rodent models, the berberine dosage was set at 100 mg/kg.

After 10 weeks of administration of intragastric berberine, body fat content was measured, and energy expenditure was assessed using indirect calorimetry. A Panlab metabolic analyzer (LE 405 Gas Analyzer) and software were used to collect and analyze basal oxygen consumption (VO_2_), and carbon dioxide production (VCO_2_) of mice. Mice were placed in metabolic cages (Oxylet Pro; Panlab) for 24 h of acclimatization, and the energy expenditure of mice was recorded for the next 24 h. Mice were kept under a 12-h light/dark cycle with free access to food and water.

The animals were weighed and anesthetized by intraperitoneal injection of pentobarbital. Following disinfection with ethanol, mice were rapidly dissected. Brown adipose tissue (BAT) was collected from the scapular region, and inguinal white adipose tissue (iWAT) was excised. Subsequently, epididymal white adipose tissue (eWAT), liver, spleen, and kidneys were isolated from the peritoneal cavity; the heart, lungs, and thymus were harvested from the thoracic cavity; and the brain was removed. A portion of the adipose tissue was fixed in paraformaldehyde for histological analysis. The remaining tissues were placed in pre-labeled nuclease-free tubes and immediately snap-frozen in liquid nitrogen and stored at − 80 °C.

### Biochemical analysis

The plasma cholesterol, high-density lipoprotein, low-density lipoprotein and triglyceride levels were measured using a biochemical analyzer (instrument model: BS-420 fully automatic biochemical analyzer; kit: Biosino Biotechnology Co., Ltd.). The expression levels of leptin, insulin, adiponectin, TNFα and IL-6 in plasma were measured using ELISA kits (Huaying Biotechnology) according to the manufacturer’s instructions.

### Cell culture

3T3-L1 preadipocytes were purchased from Peking Union Medical College (1101MOU-PUMC000155). 3T3-L1 fibroblasts were cultured in Dulbecco’s modified Eagle’s medium (DMEM) containing 10% foetal bovine serum. For the experiments, 3T3-L1 cells were cultured in 6-well plate overnight in DMEM containing 500 μM 3-isobutyl-1-methylxanthine, 0.25mM dexamethasone, and 8 μg/ml insulin and were treated with berberine (2.5 μM). The response group used siRNA to knock down TRIB1 and was treated with 2.5 μM berberine.

### Western blot analysis

Total lysates of tissues were obtained by lysis in RIPA buffer containing 1% protease inhibitor and phosphatase inhibitor. The total protein in each sample was separated by sodium dodecyl sulphate–polyacrylamide gel electrophoresis and transferred to a nitrocellulose membrane (Bio-Rad, USA). The membrane was blocked with 5% skim milk blocking buffer for 2 h at room temperature and incubated with the primary antibodies overnight at 4 °C. The membrane was then incubated with the secondary antibody for 2 h. Proteins were visualized by using S Bio-Rad imaging system (Bio-Rad, Hercules, CA, USA). L-OPA1 (~ 100 kDa) and S-OPA1 (~ 85 kDa) were quantified as separate bands; the L/S ratio was used as an index of mitochondrial fusion propensity. Table 1 lists the primary and secondary antibodies used in this study.

**Table 1:** Primary antibodies

Antibody	Company	Cat	Dilution
TRIB1	Santa Cruz	sc-393536	1:200
p-STAT3	ABclonal	AP0715	1:1000
STAT3	ABclonal	A19566	1:1000
SOCS3	ABclonal	A21981	1:1000
C/EBPα	Abcam	AB32358	1:1000
Ubi	Abmart	T55964	1:100
Secondary Antibody	Abmart	M21008	1:1000
OPA1	Proteintech	27,733-1-AP	1:1000
MFN1	Proteintech	12,186-1-AP	1:1000
DRP1	Proteintech	12,957-1-AP	1:2000
TFAM	Abcam	ab176558	1:5000
ACTIN	CST	8480	1:5000

### RNA extraction and RT-qPCR

Total RNA was isolated from brown adipocyte tissues and 3T3-L1 cells using Trizol reagent (Life, 15596026) according to manufacturer’s instructions. Total RNA concentration was determined, and cDNA was synthesized from equal RNA amounts using the indicated reverse transcription kit (RR036A; Takara Bio, Shiga, Japan), and a TB Green Premix kit (RR820A; Takara Bio) was used for real-time PCR analysis. Table 2 lists primer sequences.

**Table 2:** Primer sequences

Gene	Forward primer	Reverse primer
TRIB1	AGAACCCAGCTTAGACTGGAA	AAAAGCGTATAGAGCATCACCC
C/EBPα	CAAGAACAGCAACGAGTACCG	GTCACTGGTCAACTCCAGCAC
Leptin	GAGACCCCTGTGTCGGTTC	CTGCGTGTGTGAAATGTCATTG
LepR	GTCTTCGGGGATGTGAATGTC	ACCTAAGGGTGGATCGGGTTT
TNFα	CCCTCACACTCAGATCATCTTCT	GCTACGACGTGGGCTACAG
IL-6	TAGTCCTTCCTACCCCAATTTCC	TTGGTCCTTAGCCACTCCTTC
Pomc	ATGCCGAGATTCTGCTACAGT	TCCAGCGAGAGGTCGAGTTT
Jak2	TTGTGGTATTACGCCTGTGTATC	ATGCCTGGTTGACTCGTCTAT
Stat3	CAATACCATTGACCTGCCGAT	GAGCGACTCAAACTGCCCT
Socs3	ATGGTCACCCACAGCAAGTTT	TCCAGTAGAATCCGCTCTCCT
Ucp1	AGGCTTCCAGTACCATTAGGT	CTGAGTGAGGCAAAGCTGATTT
Dio2	AATTATGCCTCGGAGAAGACCG	GGCAGTTGCCTAGTGAAAGGT
Actin	GGCTGTATTCCCCTCCATCG	CCAGTTGGTAACAATGCCATGT

### Histology and immunostaining

The separated fat and liver tissues were fixed in 4% paraformaldehyde for more than 24 h, dehydrated with gradient ethanol (transparentized with xylene), and embedded in molten paraffin; then sliced with a slicer to obtain 4–5 μm slices, spread in a warm water bath and attached to a slide glass, and dried in a 60 °C oven overnight. During staining, the cells were dewaxed with xylene, hydrated with gradient ethanol, rinsed with running water, stained with hematoxylin for 5–7 min, rinsed with running water and differentiated with hydrochloric acid ethanol for 5–10 s, and returned to blue with running water for 15 min; stained with eosin for 1–2 min, and the staining was terminated with running water. Finally, the cells were dehydrated with gradient ethanol, transparentized with xylene, and sealed with neutral gum. This process can clearly show the lipid droplet vacuolar structure of adipocytes and the pathological changes in the fatty degeneration area of the liver.

### Bodipy staining of adipocytes

After transfection of the siRNA of Trib1 into 3T3-L1 cells, the cells were treated with 2.5 μM berberine. After 8 days, the mature Adipocytes were washed with phosphate-buffered saline (PBS) once and stained with 4 μM Bodipy staining solution (Thermo Fisher Scientific, Waltham, MA, USA) for 15 min in the dark at 37 °C. The cells were then washed twice with phosphate-buffered saline (PBS) and fixed with 4% paraformaldehyde (PFA) for 1 h. Finally, the cells were stained with Hoechst 33258 (Sigma, St. Louis, MO, USA) for 10 min and observed under a fluorescence microscope.

### Evaluation of oxidative phosphorylation in cultured adipocytes

After transfection of the siRNA of TRBI into 3T3-L1 cells, the cells were induced to differentiate and mature in 6-well plate with berberine treated or not. After 8 days, the mature adipocytes were measured the OCR and ECAR by using Seahorse XFe24 Analyzer (Agilent Technologies, Santa Clara, CA, USA).

### Statistical analysis

The results are expressed as mean ± standard error of the mean. One-way analysis of variance and Tukey’s test were used for comparisons among multiple groups with GraphPad Prism 5.0 (GraphPad Software, San Diego, CA, USA). Statistical significance was set at P < 0.05.

## Results

### Berberine alleviated hyperleptinemia and attenuated weight gain in obese mice

**Fig. 1:**
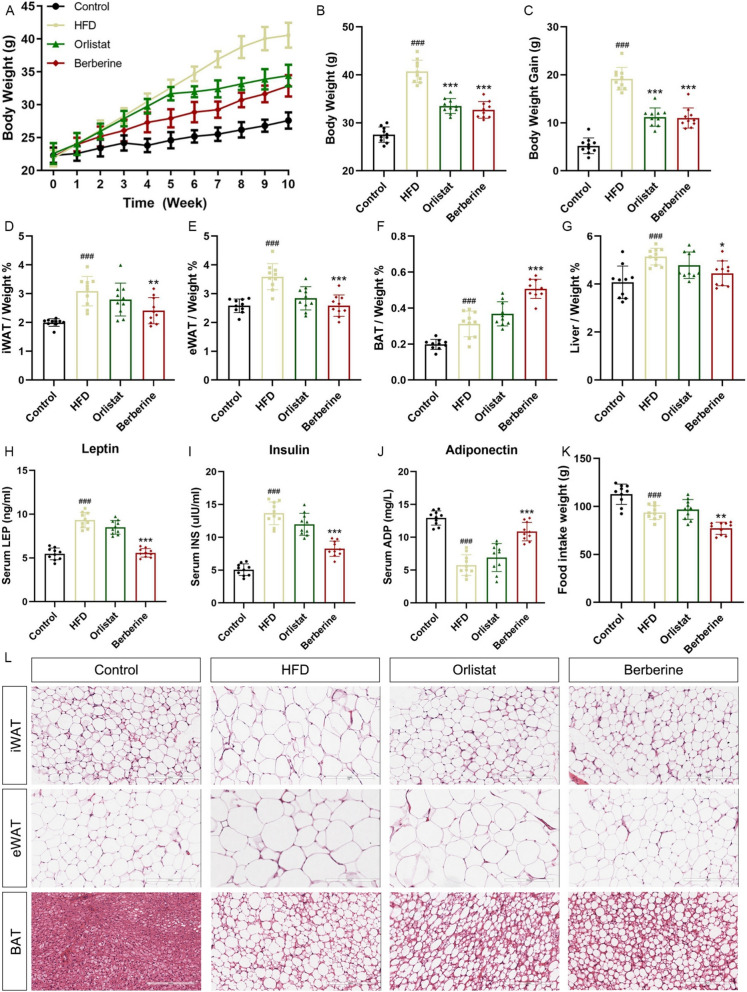
Berberine treatment significantly inhibited HFD-induced weight gain. Weight curves of mice induced by normal diet or HFD for 10 weeks (**A**), weight in the last week (**B**), and weight gain within 10 weeks (**C**). Fat mass/Liver weight-to-body weight ratio (**D**–**G**). Obesity-related serum leptin (**H**), insulin (**I**), and adiponectin (**J**) levels. Weekly food intake of mice (**K**) and representative H&E staining images of mouse iWAT, eWAT, and BAT. Scale bar: 200 μm (**L**). Data are presented as mean ± SD (n = 6). ^#^*P* < 0.05, ^##^*P* < 0.01, ^###^*P* < 0.001 *versus* the Control group; ^*^P < 0.05, ^**^P < 0.01, ^***^P < 0.001 *versus* the HFD group

C57BL/6 mice were fed a high-fat diet (HFD) to induce obesity. After 10 weeks, the body weight of the model group mice exceeded that of the control group mice by more than 30% (Fig. 1A–C), and the blood lipid levels showed significant differences (Supplementary Data 1). Mice in the treatment groups received berberine (100 mg/kg), while orlistat served as a positive control. The inguinal white adipose tissue (iWAT), abdominal white adipose tissue (eWAT), brown adipose tissue (BAT) and liver tissue of the mice were separated, and the organ coefficients were calculated (Fig. 1D–G). The results showed that berberine significantly inhibited the increase in the weight of white adipose tissue of the experimental group mice, promoted the increase of BAT in the scapula area, reduced the ectopic accumulation of fat in the liver, and reduced the body fat rate of the experimental group mice (Supplementary Data 2). In addition, we measured the key glucose and lipid metabolism factors in serum, and the results showed that berberine significantly reduced serum leptin and insulin levels and increased the expression level of adiponectin (Fig. 1H–J). Compared with the high-fat fed model group, berberine reduced the intake of mice on a HFD and suppressed appetite (Fig. 1K). H&E staining of adipose tissue showed that the area of adipocytes became smaller, and berberine promoted lipid decomposition in adipose tissue (Fig. 1L).

### Berberine improved leptin signaling in HFD-induced obese mice

**Fig. 2:**
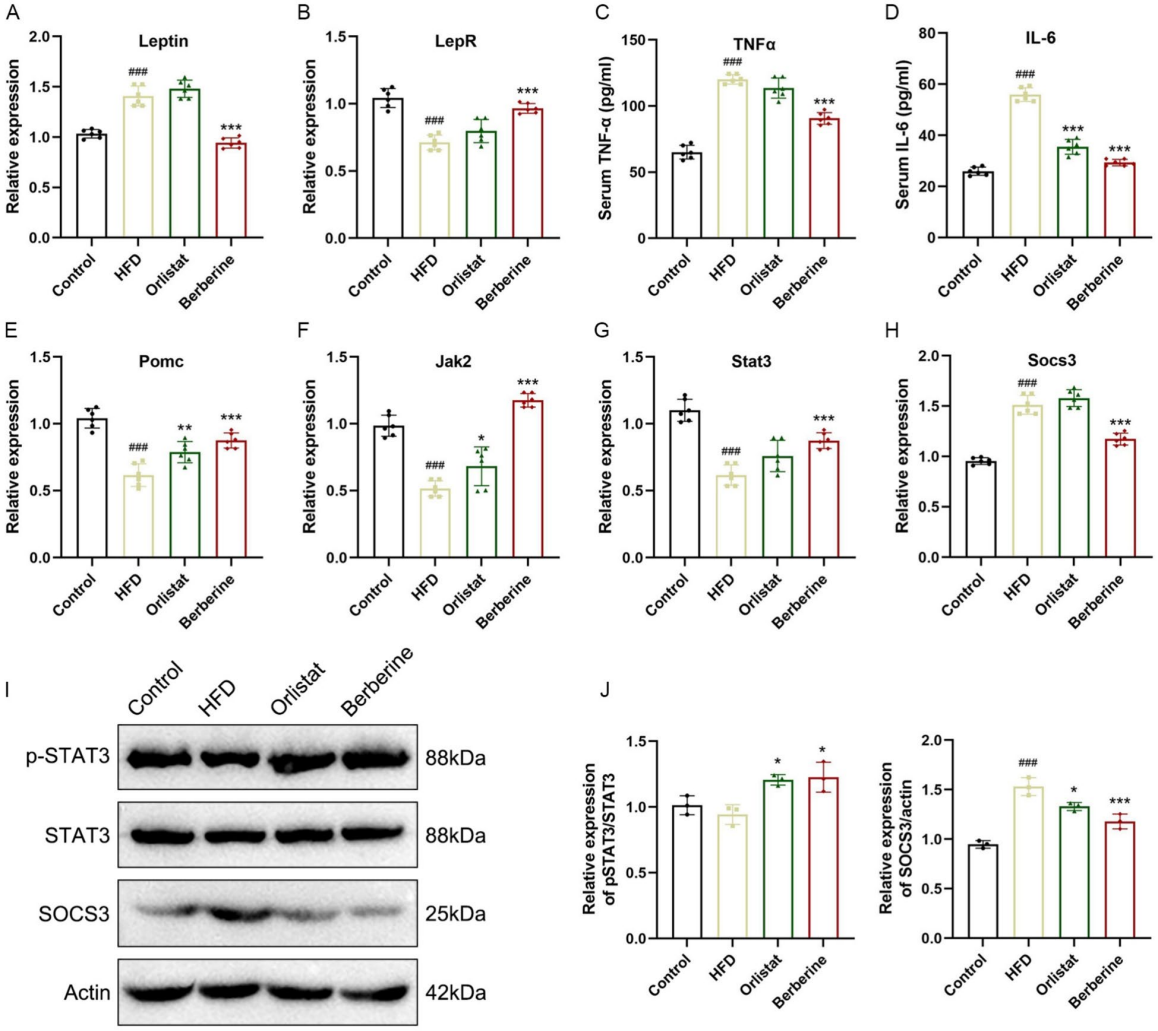
Berberine improves leptin resistance in obese mice. Adipose tissue leptin gene expression level (**A**), hypothalamic leptin receptor and inflammatory factor-related gene expression levels (**B**–**D**). Hypothalamic Pomc (**E**), Jak2 (**F**), Stat3 (**G**) and Socs3 (**H**) gene expression, as well as STAT3 protein phosphorylation ratio and SOCS3 protein expression level (**I**, **J**). Data are presented as mean ± SD (n = 6). ^#^*P* < 0.05, ^##^*P* < 0.01, ^###^*P* < 0.001 *versus* the Control group; ^*^P < 0.05, ^**^P < 0.01, ^***^P < 0.001 *versus* the HFD group

To investigate the influence of berberine on leptin signaling, the expression of key components in the Janus kinase–signal transducer and activator of transcription (JAK–STAT) pathway was examined. Berberine treatment decreased leptin mRNA levels in adipose tissue and increased the expression of leptin receptors in the hypothalamus (Fig. 2A, B). In HFD-fed mice, elevated inflammatory cytokines such as IL-6 and TNF-α are known to enhance the expression of negative regulators including SOCS3 and protein tyrosine phosphatase 1B (PTP1B), thereby impairing JAK2–STAT3 signaling. Consistent with this, TNF-α and IL-6 were elevated in the model group but were reduced by berberine treatment (Fig. 2C, D). Furthermore, we determined the expression levels of key genes and proteins in leptin signaling and leptin resistance. The results showed that berberine treatment induced the gene expression of Pomc, Jak2, and Stat3, increased the phosphorylation level of STAT3 protein, and promoted the transduction of the leptin signaling pathway (Fig. 2E–J). At the same time, berberine treatment reduced the gene and protein levels of SOCS3 and improved obesity-induced leptin resistance (Fig. 2H–J).

### Berberine increases TRIB1 expression and induces C/EBPα protein degradation

**Fig. 3:**
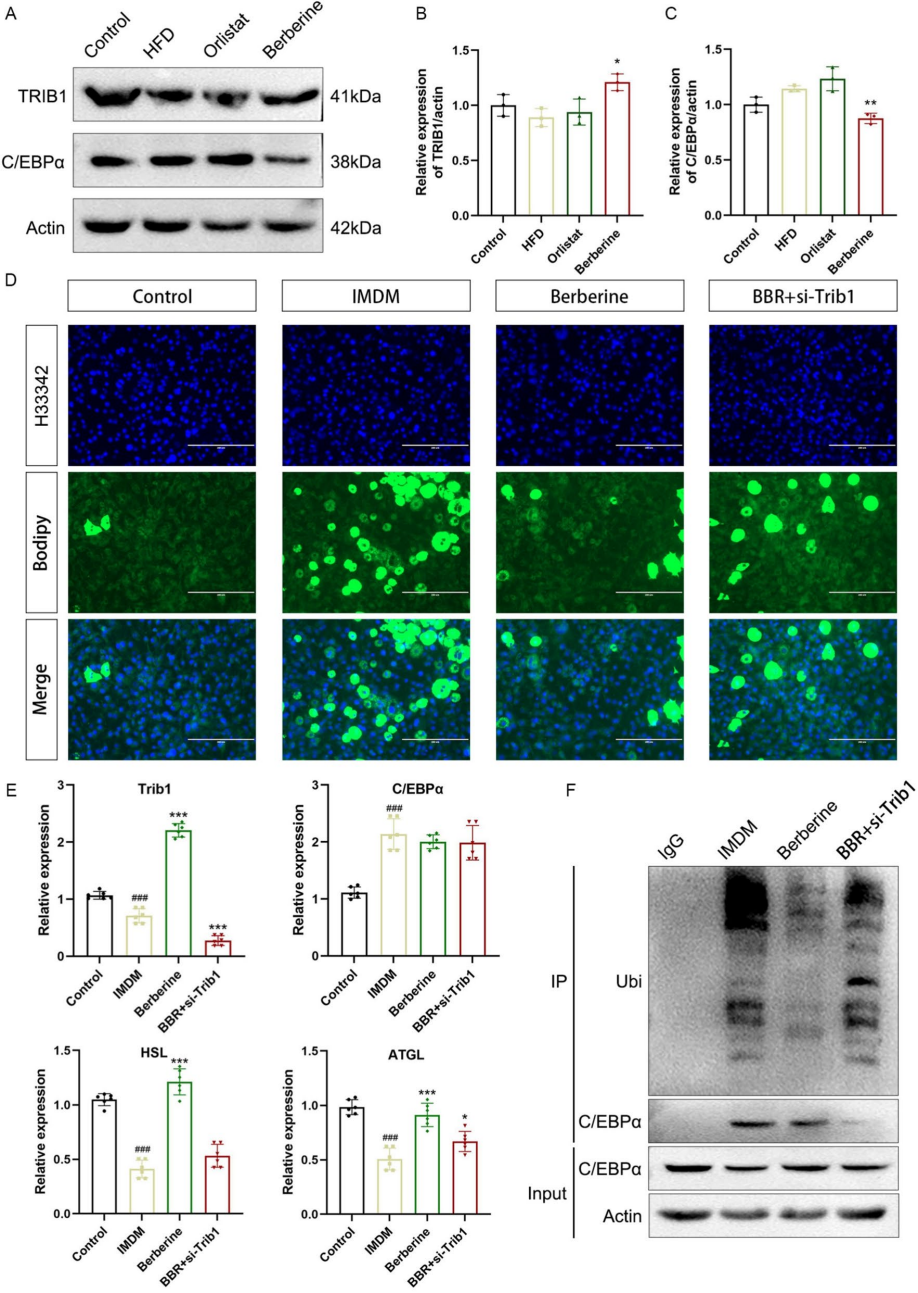
Berberine reduces lipid accumulation and induces C/EBPα protein degradation. TRIB1 and C/EBPα protein expression levels in adipose tissue (**A**–**C**). After berberine treatment and induced differentiation of 3T3-L1 cells, the H33342 and BODIPY lipid droplet staining results were obtained. Scale bar: 200 μm (**D**). TRIB1, C/EBPα and lipolysis-related HSL, ATGL gene expression levels in adipose tissue (**E**). After adipocytes were treated with berberine and MG132, immunoblotting was performed with anti-ubiquitin antibody and anti-C/EBPα antibody (**F**). Data are presented as mean ± SD (n = 3). ^#^*P* < 0.05, ^##^*P* < 0.01, ^###^*P* < 0.001 *versus* the Control group; ^*^P < 0.05, ^**^P < 0.01, ^***^P < 0.001 *versus* the induction medium and differentiation medium (IMDM) group

C/EBPα, a member of the basic leucine zipper transcription factor family, regulates adipogenesis and leptin synthesis by binding to promoter sequences of adipokine genes. Previous studies have shown that TRIB1 influences hepatic lipid accumulation through C/EBPα regulation and that berberine can enhance TRIB1 expression while reducing circulating lipid levels. Consistent with these observations, berberine treatment increased TRIB1 protein expression and decreased C/EBPα protein abundance in adipose tissue (Fig. 3A–C). In 3T3-L1 adipocytes, berberine reduced lipid accumulation, whereas this effect was blunted when TRIB1 was silenced by siRNA (Fig. 3D, E). However, it is worth noting that berberine treatment increased the gene transcription level of TRIB1 in adipocytes, but had no significant effect on the gene level of C/EBPα, suggesting that protein modification may exist (Fig. 3E). Studies have found that C/EBPα lacks the classic E3 ubiquitin ligase (COP1) binding motif, and requires the protein molecular scaffold TRIB1 to bind to C/EBPα and directly interact with COP1 through the WD40 domain of the C-terminal tail of the TRIB1 protein. We induced the differentiation of adipocytes and treated the cells with 5μM MG132, then measured the cellular ubiquitination level (Fig. 3F). The results showed that berberine treatment promoted the ubiquitination of cellular proteins, while this effect was inhibited after TRIB1 knockdown, suggesting that berberine regulates C/EBPα protein levels by increasing TRIB1 expression.

### Berberine improves hyperleptinemia and lipid accumulation through TRIB1

**Fig. 4:**
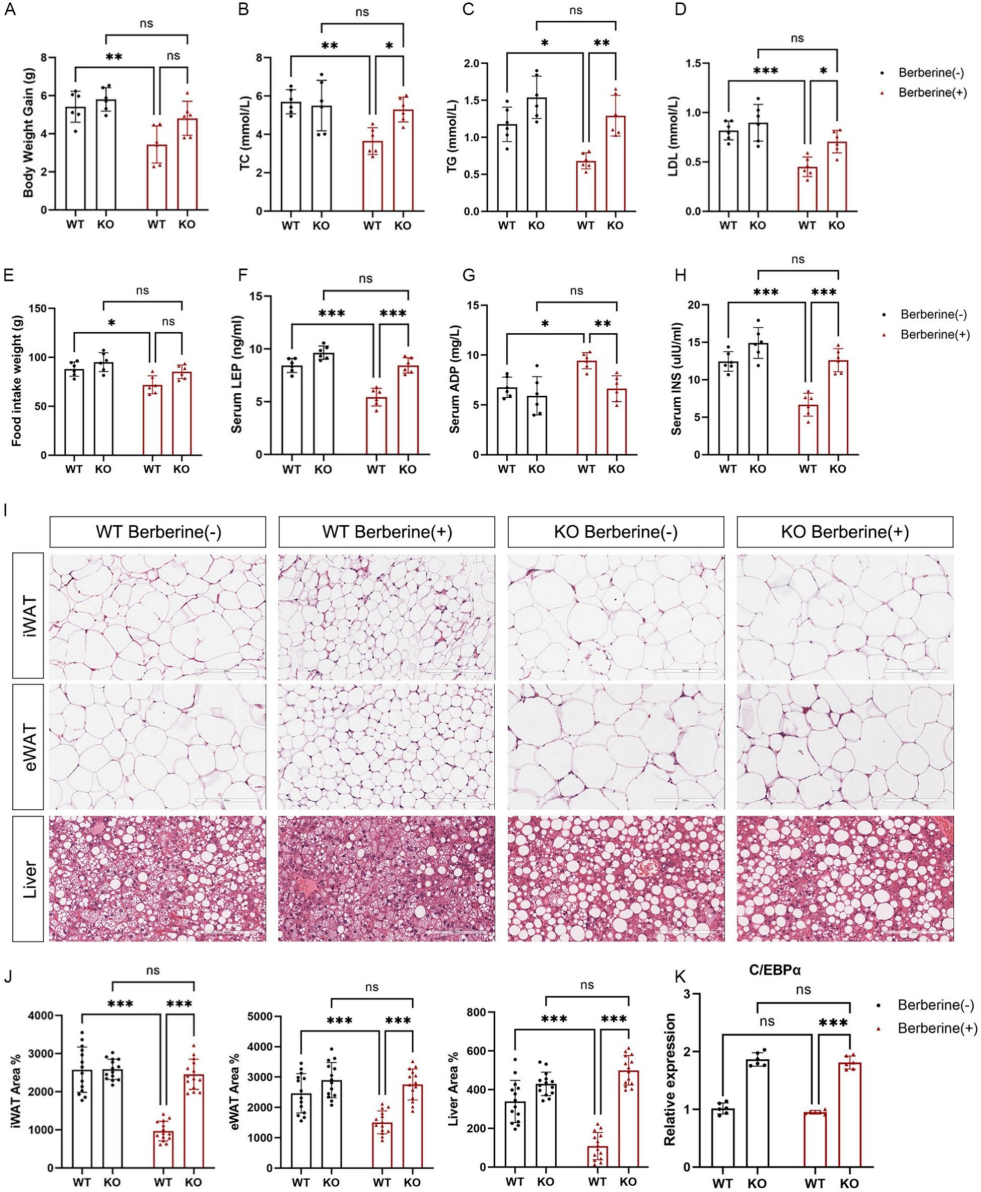
TRIB1 is a target of berberine in suppressing obesity. Weight gain (**A**), serum cholesterol (**B**), triglyceride (**C**) and low-density lipoprotein (**D**) levels of wild-type and knockout mice after 4 weeks of HFD and berberine treatment, weekly food intake (**E**), serum leptin (**F**), adiponectin (**G**) and insulin (**H**) levels. Representative H&E staining images of mouse iWAT, eWAT and Liver and statistics of lipid droplet vacuoles. Scale bar: 200 μm (**I**, **J**). Expression level of C/EBPα gene in mouse adipose tissue (**K**). Statistical method was two-way ANOVA, data are presented as mean ± SD (n = 6). ^*^P < 0.05, ^**^P < 0.01, ^***^P < 0.001 *versus* the control group

In the early stage, we established TRIB1 knockout mice, selected wild-type (WT) and knockout (KO) mice of the same litter and gave them a HFD and berberine treatment, and measured leptin levels and obesity-related phenotypes. The results showed that the effect of berberine on inhibiting mouse weight gain was suppressed in TRIB1 knockout mice (Fig. 4A), and TRIB1 knockout mice treated with berberine still showed increased TC, TG and LDL (Fig. 4B–D), and the effect of reducing food intake was also suppressed (Fig. 4E). Compared with WT mice, berberine treatment improved hyperleptinemia, while the effect of berberine was eliminated in TRIB1 knockout mice (Fig. 4F). Similarly, the improvement of berberine on key factors of glucose and lipid metabolism was also suppressed due to the knockout of TRIB1 (Fig. 4G, H). Adipose tissue and liver tissue were sliced to observe changes in lipid accumulation. The results showed that berberine could effectively reduce lipid accumulation, reduce the area of adipocytes and improve the area ofliver vacuoles in WT mice, but the benefits of berberine were eliminated by knockout of TRIB1 (Fig. 4I, J). These results suggest that TRIB1 is an important target of berberine in inhibiting weight gain, and its function is achieved by regulating leptin levels through C/EBPα (Fig. 4K).

### Berberine improves the metabolic level of obese mice through TRIB1

**Fig. 5:**
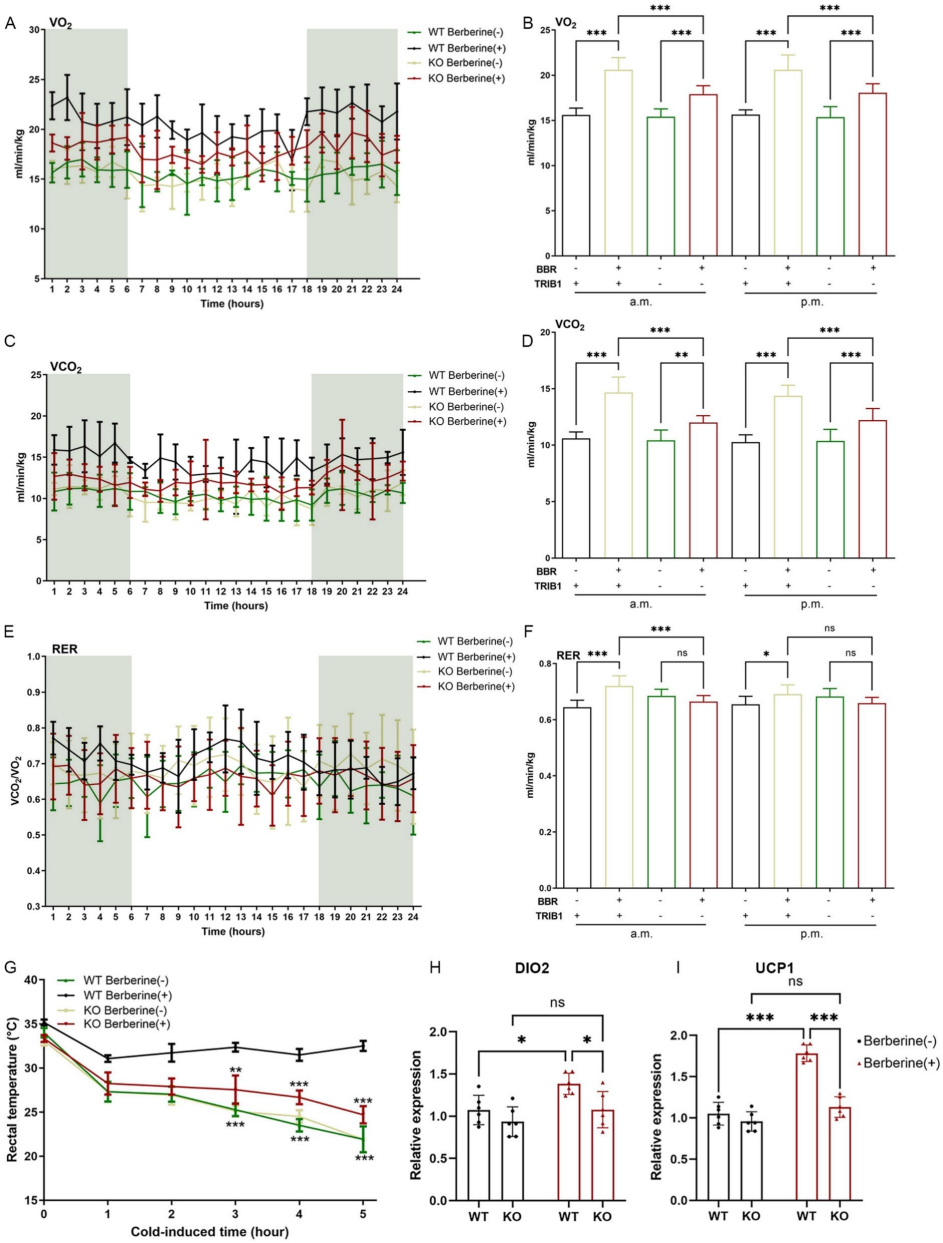
Berberine improves the metabolic level of obese mice through TRIB1. After wild-type and knockout mice were treated with HFD and berberine for 4 weeks, energy expenditure was evaluated by measuring oxygen consumption (**A**, **B**), carbon dioxide release (**C**, **D**) and respiratory quotient (**E**, **F**). After 5 h of cold exposure, the rectal temperature of the mice was measured (**G**). Expression levels of thermogenic genes DIO2 and UCP1 in mouse adipose tissue (**H**, **I**). Two-way ANOVA was used for statistical analysis, and data are presented as mean ± SD (n = 6). ^*^P < 0.05, ^**^P < 0.01, ^***^P < 0.001 *versus* the control group

Energy expenditure was assessed using the Comprehensive Lab Animal Monitoring System (CLAMS). In WT mice, berberine treatment increased oxygen consumption and carbon dioxide production throughout a 12-h light/dark cycle compared with HFD controls. Although TRIB1-KO mice treated with berberine also showed a mild increase in oxygen consumption, their respiratory metabolism remained significantly lower than that of WT mice receiving berberine (Fig. 5A–D). The respiratory quotient was reduced in all HFD-fed groups, reflecting increased fat oxidation under insulin-resistant conditions. Berberine treatment improved insulin sensitivity and partially normalized substrate utilization in WT mice, but this effect was dependent on TRIB1 (Fig. 5E, F). To evaluate adaptive thermogenesis, mice were exposed to cold, and rectal temperature was monitored hourly (Fig. 5G). WT mice treated with berberine exhibited higher cold-induced thermogenic capacity, whereas this response was largely absent in TRIB1-KO mice. Expression of key thermogenic genes, including *Dio2* and *Ucp1*, followed the same pattern (Fig. 5H, I). Together, these data suggest that TRIB1 is required for the enhancement of energy expenditure and thermogenesis induced by berberine.

### Berberine improved mitochondrial function via TRIB1-associated regulation of dynamics

**Fig. 6:**
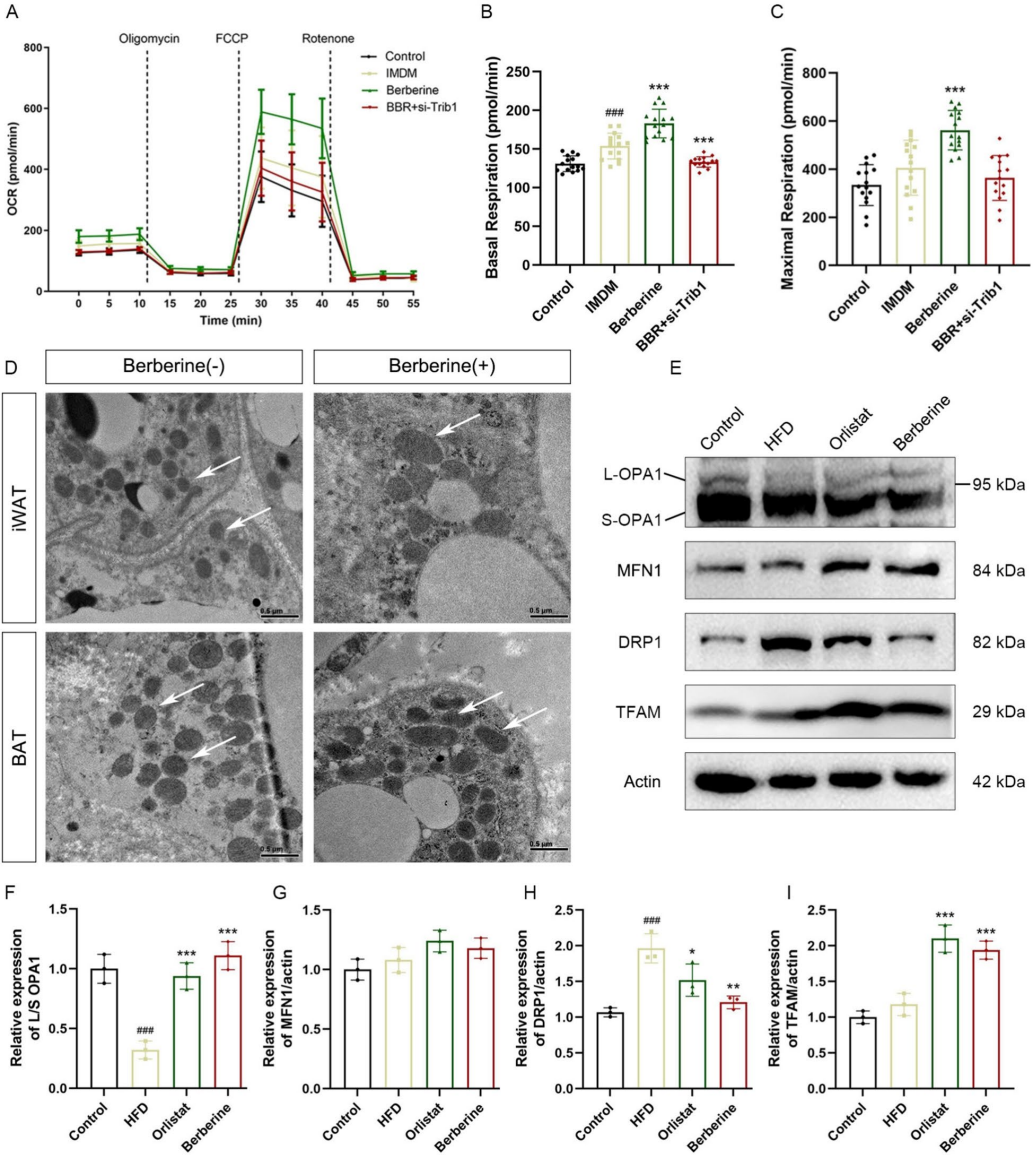
Berberine treatment promotes mitochondrial fusion and improves mitochondrial fragmentation. After berberine and siRNA treatment, 3T3-L1 adipocytes were induced to differentiate, and the respiratory metabolism level of cells was measured (**A**–**C**). Transmission electron microscopy was used to observe the mitochondrial morphology in iWAT and BAT. Scale bar: 0.5 μm (**D**). Protein expression levels of mitochondrial fusion proteins OPA1 and MFN1, mitochondrial fission protein DRP1 and mitochondrial transcription factors in adipose tissue (**E**–**I**). Data are presented as mean ± SD (n = 6). ^#^*P* < 0.05, ^##^*P* < 0.01, ^###^*P* < 0.001 *versus* the Control group; ^*^P < 0.05, ^**^P < 0.01, ^***^P < 0.001 *versus* the HFD group

After inducing differentiation and incubating drugs in 3T3-L1 cells, 1 × 10^5^ cells were counted per well and the respiratory metabolic level of cells was measured by Seahorse Bioscience. The results showed that berberine treatment increased the basal respiratory metabolic level of cells, and the maximum respiratory metabolic level of the berberine-treated group was significantly increased after the addition of FCCP, indicating that berberine enhanced the mitochondrial function of adipocytes, and the knockdown of TRIB1 eliminated these benefits (Fig. 6A–C). Furthermore, we used transmission electron microscopy to observe the iWAT and BAT of mice in the model group and berberine-treated group. The results showed that the mitochondria in the adipose tissue of obese mice in the model group were small, short and round, and berberine treatment improved the abnormal mitochondrial morphology in adipose tissue (Fig. 6D). Because mitochondrial function depends on the balance between fission and fusion, the expression of key regulatory proteins was analyzed. Compared with the HFD group, berberine treatment elevated the levels of the fusion-related proteins L-OPA1 and MFN1, while reducing expression of the fission protein DRP1 (Fig. 6E–I), These molecular changes coincided with improved mitochondrial integrity and overall metabolic function. Collectively, these results suggest that berberine enhanced mitochondrial quality and oxidative capacity through TRIB1-associated regulation of mitochondrial dynamics.

## Discussion

One of the main findings of this study was that berberine reduced leptin synthesis and secretion by upregulating TRIB1 expression in adipose tissue and promoting the ubiquitination and degradation of C/EBPα. This mechanism provides a plausible explanation for the improvement of hyperleptinemia in obese mice. Using TRIB1 knockout mice, we found that the metabolic benefits of berberine, such as reduced body-weight gain, decreased fat accumulation, and improved leptin sensitivity, were largely dependent on TRIB1. Berberine also restored hypothalamic leptin signaling by enhancing JAK2–STAT3 phosphorylation and reducing SOCS3 expression, thereby mitigating leptin resistance. These data suggest that berberine regulates leptin production and signaling through the TRIB1–C/EBPα axis, providing mechanistic insight into its anti-obesity effects.

Beyond leptin regulation, berberine improved mitochondrial function and energy expenditure through TRIB1. Previous work showed that TRIB1 is essential for BAT thermogenesis and mitochondrial respiration [7]. In the present study, berberine increased TRIB1 expression and improved mitochondrial morphology, characterized by enhanced OPA1 and MFN1 expression and reduced DRP1 activity. On the one hand, a large number of studies have shown that leptin can inhibit the expression of mitochondrial fission protein DRP1 and inhibit mitochondrial fragmentation [11, 14]. On the other hand, in obesity-related gallbladder cancer, researchers found that leptin induces mitochondrial fusion [15], and Mann et al. also found that leptin secretion is closely related to mitochondrial fusion [16]. Our study found that berberine treatment can increase TRIB1 expression and improve leptin resistance, thereby adjusting the ratio of OPA1 fusion-promoting form (long, about 100 KDa) and non-fusion-promoting form (short, about 85 KDa), restoring the unbalanced mitochondrial dynamics in obesity, thereby improving the overall quality and function of mitochondria. Together, these findings highlight a role for TRIB1 in linking leptin signaling to mitochondrial dynamics and energy metabolism, suggesting a pathway by which berberine promotes lipid oxidation and thermogenesis.

Current studies have supported the role of berberine in the treatment of metabolic diseases such as non-alcoholic fatty liver disease, obesity and diabetes, among which the main molecular targets involved include LDLR, AMPK and PPAR [17]. Our findings add TRIB1 as an additional mediator of its anti-obesity actions. By facilitating C/EBPα ubiquitination and degradation, berberine may downregulate adipogenic gene expression, thereby limiting adipose expansion [18]. Previous researchers have found that TRIB1 can recruit C/EBPα to the ubiquitin ligase COP1 to promote ubiquitination [19], thereby regulating liver lipogenesis and blood lipid levels [4]. Singh et al. found that berberine reduced plasma triglyceride levels and upregulated liver TRIB1 [20] in LDLR-deficient mice, suggesting that berberine has an LDLR-independent lipid-lowering mechanism. Furthermore, berberine reduced circulating insulin and pro-inflammatory cytokines (TNF-α and IL-6) while increasing adiponectin levels, reflecting an improved systemic metabolic profile. Combined with previous evidence that berberine activates AMPK and suppresses SREBP1c, our results suggest that its metabolic benefits arise from multi-targeted synergistic regulation rather than a single pathway.

In summary, this study indicated that the pseudokinase TRIB1 acts as a key mediator linking berberine to improvements in leptin sensitivity, lipid metabolism, and mitochondrial function. Through the TRIB1–C/EBPα axis, berberine coordinates leptin signaling, adipogenesis, and mitochondrial remodeling to enhance systemic metabolic efficiency. While TRIB1 emerges as a promising molecular node in energy regulation, further investigation is warranted to assess its therapeutic potential.

Although our results provide mechanistic insights into the TRIB1–C/EBPα axis underlying berberine’s anti-obesity effects, several limitations should be noted. First, the study was conducted in a rodent model with relatively small sample sizes, and leptin signaling differs substantially between rodents and humans, which may limit direct translational relevance. Second, berberine exhibits low bioavailability and complex pharmacokinetics, potentially constraining its efficacy in human applications. Third, the upstream pathways through which berberine upregulates TRIB1 remain to be elucidated. Future studies employing transcriptomic and receptor-targeted approaches will be required to clarify how berberine engages TRIB1 regulation. Addressing these limitations will refine the mechanistic framework and inform rational design of TRIB1-targeted interventions.

## Supplementary Information

Additional file 1

## Data Availability

The datasets generated in this study are available from the corresponding author upon request.

## Abbreviations

TRIB1: Tribbles homolog 1
C/EBPα: CCAAT enhancer-binding protein alpha
BAT: Brown adipose tissue
iWAT: Inguinal white adipose tissue
eWAT: Epididymal white adipose tissue
UCP1: Uncoupling protein 1
TG: Triglyceride
TC: Total cholesterol
LDL: Low-density lipoprotein
RT-qPCR: Quantitative real-time polymerase chain reaction
IMDM: Induction medium and differentiation medium
VO_2_: Oxygen consumption
VCO_2_: Carbon dioxide production
RQ: Respiratory quotient
DRP1: Dynamin-related protein 1
MFN1: Mitofusin 1
OPA1: Optic atrophy 1
DMEM: Dulbecco’s modified Eagle’s medium
FBS: Fetal bovine serum
PBS: Phosphate-buffered saline
OCR: Oxygen consumption rate
ECAR: Extracellular acidification rate
PFA: Paraformaldehyde
IBMX: 3-Isobutyl-1-methylxanthine
KO: Knockout
WT: Wild-type
HFD: High-fat diet
NAFLD: Non-alcoholic fatty liver disease
β3-AR: β3-Adrenergic receptor
