# Supplementary figures and images for: Molecular mechanism of berberine in ameliorating leptin resistance and mitochondrial dysfunction through the TRIB1-C/EBPα axis in obesity
Source: Chin Med. 2026 Jan 26;21:55. doi: 10.1186/s13020-025-01296-7 (PMC12833932; doi:10.1186/s13020-025-01296-7)

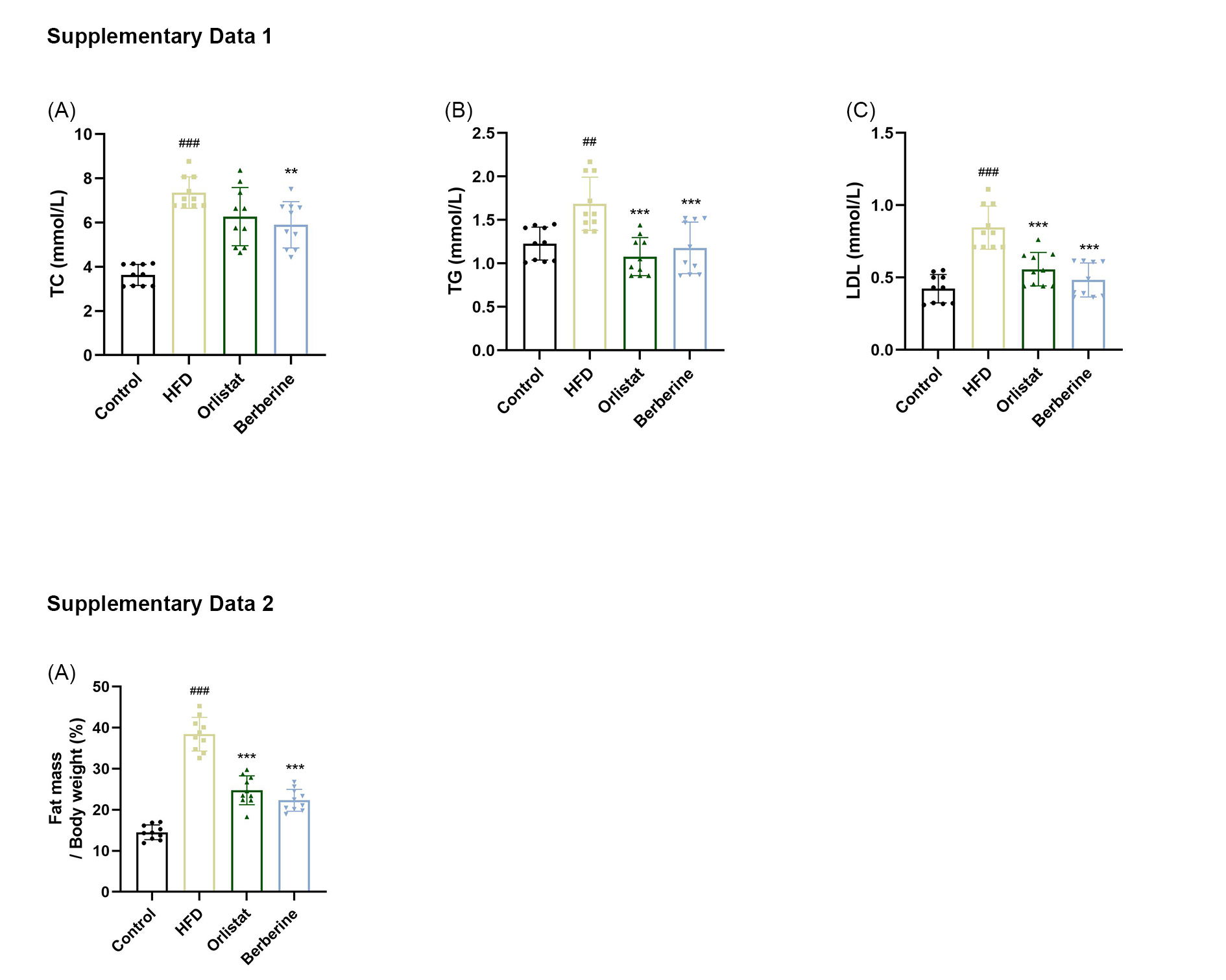

Supplement: Supplementary file 1 — Additional file 1 [file 13020_2025_1296_MOESM1_ESM.jpg]
